# New progress in the production, oxidative damage, and scavenging mechanisms of reactive oxygen species in plants under abiotic stress

**DOI:** 10.3389/fpls.2026.1774033

**Published:** 2026-03-06

**Authors:** Ranran Liu, Shulei Wang, Jie Song

**Affiliations:** 1 College of Agriculture and Biology, Liaocheng University, Liaocheng, China; 2 Key Laboratory of Ecological Safety and Sustainable Development in Arid Lands, Xinjiang Institute of Ecology and Geography, Chinese Academy of Sciences, Urumqi, China; 3 College of Life Science, Shandong Normal University, Jinan, China

**Keywords:** abiotic stress, antioxidant mechanisms, plant adaptation, redox homeostasis, ROS detection techniques

## Abstract

Reactive oxygen species (ROS) are central players in plant abiotic stress responses, functioning as both toxic byproducts and vital signaling molecules. Under normal physiological conditions, ROS participate in the regulation of plant growth and development. However, under stress conditions, ROS metabolism exhibits remarkable stress-specificity, leading to either adaptive signaling or oxidative damage. A comparative understanding of these distinct patterns is critical for advancing stress tolerance engineering. This review systematically elaborates on the mechanisms of ROS production under various abiotic stresses, their dual roles in signaling and oxidative damage, and the corresponding multilayer antioxidant adaptations in plants. We place particular emphasis on comparing the characteristic ROS signatures and regulatory networks triggered by drought, salinity, extreme temperatures, heavy metals, ultraviolet radiation and ozone. Furthermore, we summarize cutting-edge technologies for *in vivo* ROS detection that are revolutionizing the spatiotemporal understanding of ROS dynamics, these advanced tools enable real-time, subcellular resolution of ROS production, scavenging, and signaling processes, thereby propelling the mechanistic dissection of plant redox homeostasis under stress. Ultimately, we highlight how plants achieve acclimation by precisely orchestrating the “double-edged sword” nature of ROS through an integrated regulatory network. This synthesis not only consolidates the mechanistic understanding but also offers a strategic perspective for designing crops with tailored ROS regulatory capacities to enhance resilience in a changing climate.

## 1 Introduction

The continuous growth of the global population, coupled with the escalating impacts of climate change, pose a serious threat to food security. During their life cycle, plants encounter a variety of abiotic stresses, such as drought, salinity, and extreme temperatures, heavy metals, and ultraviolet radiation. These stressors significantly constrain crop growth and yield, with profound implications for agricultural productivity worldwide.

Within the complex signaling network of plant stress responses, reactive oxygen species (ROS) function as central signaling hubs (Mittler et al., 2004). Under normal physiological conditions, ROS are primarily generated through the mitochondrial electron transport chain (ETC), chloroplast photosynthesis, and plasma membrane NADPH oxidases (RBOHs), with basal levels maintained at low concentrations (Mittler, 2002; Miller et al., 2010). Although traditionally considered harmful metabolic byproducts, ROS are now recognized as crucial signaling molecules at low concentrations that regulate plant growth, hormone signaling, and environmental acclimation (Mittler et al., 2022). As inevitable byproducts of aerobic metabolism in organelles such as chloroplasts and mitochondria (Mittler et al., 2011), ROS, under homeostasis, serve as essential secondary messengers in the processes including stomatal movement, programmed cell death (PCD), and systemic acquired resistance (SAR) (Torres and Dangl, 2005; Mittler et al., 2022). However, under stress conditions, ROS production increases dramatically, disrupting cellular homeostasis and inducing oxidative stress (Mittler et al., 2004, 2011). Due to their strong oxidative capacity, excessively accumulated ROS attack critical biomolecules, including proteins, lipids, and nucleic acids, causing structural damage and metabolic dysfunction that ultimately inhibits plant growth and can lead to cell death (Mittler, 2002; Choudhury et al., 2017). To mitigate these adverse effects, plants have evolved sophisticated antioxidant defense systems comprising enzymatic components such as superoxide dismutase (SOD) and catalase (CAT), and non-enzymatic antioxidants including ascorbate (AsA) and glutathione (GSH), which work in concert to maintain ROS homeostasis and mitigate oxidative damage (Gill and Tuteja, 2010). Therefore, precise regulation of ROS homeostasis is essential for plant adaptation and survival.

Recent years, significant advances in elucidating the mechanisms of ROS production, signaling transduction, and antioxidant regulatory in plants have been witnessed under stress conditions. Building on recent advances, this review systematically examine ROS production pathways analyze the dual roles of ROS as signaling molecules and agents of oxidative damage, elaborate on the intricate antioxidant regulatory networks deployed under different stresses, summarizes stress-specific ROS metabolic features, and discusses emerging detection technologies and future research priorities. This synthesis provides a new perspective on plant ROS metabolism under abiotic stress, aiming to offer theoretical support for developing stress-tolerant crops and sustainable agricultural systems.

## 2 Generation and initial fates of ROS under abiotic stress

Under abiotic stress conditions, ROS generation is significantly enhanced across various subcellular compartments, primarily driven by disruptions in electron transport chains and metabolic imbalances. While the fundamental sources of ROS (chloroplasts, mitochondria, peroxisomes, and plasma membrane) remain consistent, the specific triggers, predominant sites, and intensity of ROS production are often stress-dependent.

### 2.1 Primary sites and pathways of ROS production

ROS, including superoxide anion (O_2_•^-^), hydrogen peroxide (H_2_O_2_), hydroxyl radical (•OH), and singlet oxygen (¹O_2_), are byproducts of aerobic metabolism in various subcellular compartments. They exhibit distinct chemical properties, reactivity, half-lives, and mobility, which underlie their diverse biological roles (Mittler, 2002). While their chemical reactivity and biological functions vary, recent advances highlight that their production under stress is governed by a set of conserved regulatory principles and molecular modules, rather than being merely stochastic leakage (Schieber and Chandel, 2014; Castro et al., 2021). Understanding the inherent production mechanisms at these sites is crucial for deciphering how their dysregulation under stress leads to distinct ROS signatures.

Chloroplasts are identified as major sites of ROS production. During photosynthesis, both Photosystem II (PSII) and Photosystem I (PSI) can produce O_2_•^-^ and ¹O_2_ via electron leakage, with O_2_•^-^ being subsequently converted to H_2_O_2_ by SOD (Asada, 2006). Under stress conditions, an imbalance between the light reactions and carbon assimilation causes over-reduction of the electron transport chain, dramatically exacerbating chloroplastic ROS production (Mittler, 2002; Asada, 2006).

Mitochondria contribute significantly via electron leakage at Complex I and III of the respiratory ETC, thereby forming O_2_•^-^ (Mittler, 2002; Huang et al., 2016). Stresses such as low temperature can impair mitochondrial ETC function, further enhancing this source of ROS (Huang et al., 2016).

Plasma membrane-localized RBOHs are key enzymatic ROS sources.: These enzymatic sources produce O_2_•^-^ via NADPH-dependent oxygen reduction, with subsequently dismutated to H_2_O_2_ (Miller et al., 2009). The activity of RBOHs is rapidly activated by stress signals, often through calcium binding and phosphorylation events, making it a crucial amplifier for the early ROS burst (Miller et al., 2009). Notably, phosphorylation of RBOHs serves as an initial trigger in the Ca²^+^-ROS signaling network (Kimura et al., 2012).

Peroxisomes generate ROS primarily during fatty acid β-oxidation (Mittler et al., 2004). Additionally, Fenton and Haber-Weiss reactions in the cytoplasm can produce highly reactive •OH, particularly in the presence of redox-active metals (Mittler, 2002; Mittler et al., 2004). Collectively, these spatially organized pathways form the fundamental infrastructure for ROS production. Abiotic stresses typically do not create novel sources but rather dysregulate and amplify these pre-existing mechanisms in a stress-specific manner, leading to distinct spatiotemporal ROS signatures.

In summary, these spatially organized pathways form the fundamental infrastructure for ROS production. Abiotic stresses generally do not create new sources but specifically dysregulate and amplify these pre-existing mechanisms, leading to distinct spatiotemporal ROS signatures.

### 2.2 Stress-specific ROS production

Different abiotic stresses selectively engage specific ROS production pathways. This generates unique spatiotemporal signatures that critically influence physiological outcomes, as detailed in the following sections and **Table 1**.

**Table 1:** ROS production and antioxidant defense mechanisms in plants under various stress conditions.

Stress type	Key ROS sources/pathways	Antioxidant response	Associated signaling/regulatory factors	References
Drought	Chloroplasts/Mitochondria/PM: • ETC leakage. • Stomatal closure →reduced CO_2_ assimilation → photoinhibition PM: • RBOHs (e.g., RbohD)-mediated ROS burst.	Enzymatic: ↑ SOD, CAT, APX, POD activities. Non-Enzymatic: Accumulation of AsA, GSH, flavonoids. Metabolic: ABA-induced antioxidant activation.	Hormones: ABA, BRs. Protein Interactions: • *TaWD40-4B.1^C^* interacts with & stabilizes TaCAT (Wheat). • CPK8 phosphorylates CAT3 (*Arabidopsis*). • E3 ligase CIRP1 degrades CAT2/CAT3.	Zou et al., 2015; Tian et al., 2023; Chen et al., 2025; Yao et al., 2025.
Salt Stress	Chloroplasts/Mitochondria: • Ion toxicity disrupts ETC → electron leakage. Peroxisomes: • Enhanced photorespiration (especially in C3 plants) → H_2_O_2_ PM: • RBOHs (e.g., RBOHD/F) generate O_2_•^-^. Apoplast: • Polyamine oxidation → H_2_O_2_.	Enzymatic: Significant ↑ SOD, POD, CAT, APX activities. Halophytes: Stronger & faster enzyme activation. Transcriptional: WRKY, bZIP, NTL TFs activate antioxidant genes.	Hormones: ABA (forms a positive feedback loop with ROS). Regulatory Modules: • LBD11-PRX71-RBOHD/F (*Arabidopsis*). • SpsWRKY53–SpsMDP1–SpsPP2C80 (*S. psammophila*). • GmNTL1 activates *GmRbohBs* (Soybean). • OsCPK12 phosphorylates OsCATA/C (Rice).	Dang et al., 2025; Li et al., 2025; Wang et al., 2024; Zhang et al., 2023a, b.
High Temperature	Chloroplasts: • Damage to PSII & PETC → O_2_•^-^, ¹O_2_. Mitochondria: • Impaired ETC. PM: • RBOHs (e.g., OsRbohB) amplify ROS. Endoplasmic Reticulum: • Protein misfolding & UPR.	Enzymatic: Activation of HSFs/HSPs; altered SOD, CAT, APX activities. Non-Enzymatic: Volatile isoprenoids scavenge ROS or act as signals. PTM: CPK28 phosphorylates APX2.	Signaling: Ca²^+^-CaM-CDPK-MAPK cascade; ABA-SAPK2 pathway. Regulators: • “*AcEGY3*-CSD2-H_2_O_2_-HSFA2-2” positive feedback loop (Kiwifruit). ZmCDPK7 phosphorylates RBOHB (Maize).	Ling et al., 2025; Wang et al., 2025; Zuo et al., 2025; Zhao et al., 2023a; Hu et al., 2021
Low Temperature	Chloroplasts: • Reduced thylakoid fluidity & Calvin cycle inhibition → excess light energy → ¹O_2_, O_2_•^-^. Mitochondria: • Dysfunctional ETC Complex I → O_2_•^-^ (regulated by e.g., SOP10 in rice).	Enzymatic: General ↑ SOD, APX, DHAR, MDHAR, GR activities. Non-Enzymatic: Accumulation of anthocyanins, proanthocyanidins, AsA. Metabolic: Increased ω-3 fatty acids maintain membrane fluidity	Epigenetic: • DgMYB-DgATX module activates *DgPOD* via H3K4me3 (*Chrysanthemum*). • PtrbHLH activates *PtrCAT* (Pummelo). • AcePosF21 interacts with AceMYB102 to promote AsA synthesis (Kiwifruit) Transcriptional: MYB, bHLH, bZIP TFs activate	Luo et al., 2024; Zhang et al., 2024a; Liu et al., 2023; Geng et al., 2019
Heavy Metals	Direct (Cu, Cr): • Catalyze ·OH production via Fenton/Haber-Weiss reactions. Indirect (Cd, Pb): • Disrupt ETC, displace cofactors → O_2_•^-^. General: Persistent and cumulative oxidative stress.	Enzymatic: ↑ SOD, CAT, APX, GST activities. Non-Enzymatic: Accumulation of GSH, phytochelatins, flavonoids, glucosinolates. Exogenous: SiO_2_ nanoparticles upregulate antioxidant defense.	Systemic Signaling: RBOH-ROS-Auxin cascade reshapes root architecture (Rice).	Fu et al., 2024; Malik et al., 2025; Wang et al., 2023;
UV-B Radiation	Chloroplasts: • Direct damage to D1/D2 proteins; inhibition of Rubisco & Calvin cycle → over-reduction of ETC → O_2_•^-^, ¹O_2_.	Enzymatic: ↑ SOD, POD, CAT activities and gene expression (e.g., *POX, GST*). Non-Enzymatic: Significant accumulation of flavonoids and phenolics as UV screens & antioxidants.	Photomorphogenesis: Activation of the UVR8 signaling pathway. Gene Expression: Increased transcriptional activity of antioxidant enzyme genes.	Ma et al., 2019; Kirova et al., 2024; Wang et al., 2023
Ozone (O_3_)	Apoplast: • O_3_ decomposition → O_2_•^-^, H_2_O_2_. Chloroplasts/Mitochondria: • Secondary ROS sources mediating stomatal closure.	Enzymatic: APX, MDHAR, GR activities increase then may decline. Non-Enzymatic: Apoplastic AsA is first line of defense; AsA-GSH cycle can be depleted. Exogenous: EDU & bio-silver nanoparticles (B-AgNPs) enhance capacity.	Stomatal Regulation: SLAC1, OST1 control stomatal movement. Post-transcriptional: O_3_ rapidly induces miRNA-mediated reprogramming.	Kannaujia et al., 2024; Wang et al., 2011; Vahisalu et al., 2010

PM, Plasma Membrane; ETC, Electron Transport Chain; RBOH, Respiratory Burst Oxidase Homolog; PTI/ETI, Pattern-/Effector-Triggered Immunity; HAMPs, Herbivore-Associated Molecular Patterns; TFs, Transcription Factors; PTM, Post-Translational Modification; AsA, Ascorbate; GSH, Glutathione; SOD, Superoxide Dismutase; CAT, Catalase; APX, Ascorbate Peroxidase; POD, Peroxidase; GR, Glutathione Reductase; HSFs/HSPs, Heat Shock Factors/Proteins; MDHAR, Monodehydroascorbate Reductase; DHAR, Dehydroascorbate Reductase.

#### 2.2.1 Drought stress

Drought stress, a major constraint to global agricultural productivity (Dai, 2013), primarily induces ROS overproduction through stomatal closure. This closure restricts CO_2_ influx, inhibits the Calvin cycle, and leads to over-reduction of the PETC, thereby stimulating O_2_•^-^ and ¹O_2_ production in chloroplasts (Asada, 2006). Concurrently, drought-induced metabolic shifts and impaired mitochondria respiration can also contribute to increased electron leakage and O_2_•^-^ production (Choudhury et al., 2017). When ROS accumulation surpasses cellular scavenging capacity, oxidative stress occurs, fundamentally impairing normal plant growth (Choudhury et al., 2017). Furthermore, plasma membrane RBOHs are rapidly activated by drought-induced osmotic stress and ABA signaling, leading to a prominent apoplastic ROS burst that mediates in stomatal closure and stress signaling (Mittler et al., 2022). The excessive ROS accumulation resulting from theses mechanisms is a major contributor of growth inhibition under drought conditions.

#### 2.2.2 Salt stress

Salt stress induces a rapid burst of ROS primarily through two interconnected pathways, including ionic toxicity and osmotic stress. Elevated intracellular Na^+^ accumulation directly impairs the function of photosynthetic and respiratory electron transport chains in chloroplasts and mitochondria, leading to increased electron leakage and O_2_•^-^ production (Hernandez et al., 2001; Bose et al., 2014). In chloroplasts, Na^+^ inhibits PSII activity and limits NADP^+^ regeneration, thereby enhancing the Mehler reaction. In mitochondria, over-reduction of the electron transport chain under salt stress can lead to increased O_2_•^-^ generation even through the alternative oxidase (AOX) pathway (Huang et al., 2016; Miller et al., 2010). Meanwhile, Na^+^ competes with Ca²^+^ for binding to signal molecules such as calmodulin, disturbing intracellular calcium signal homeostasis and indirectly activating RBOH-mediated ROS production (Bose et al., 2014; Kimura et al., 2012). Salt stress also promotes apoplastic polyamine oxidation, yielding further H_2_O_2_ via polyamine oxidases (Rodríguez et al., 2009). The osmotic component of salt stress, similar to drought, inducing stomatal closure and photoinhibition. In C3 plants, this enhances photorespiration, leading to substantial H_2_O_2_ production in peroxisomes via glycolate oxidase. By contrast, C4 plants, with their CO_2_-concentrating mechanisms, are less prone to such photorespiratory ROS bursts, highlighting a key physiological distinction between photosynthetic types (Stepien and Klobus, 2005; Bose et al., 2014). Moreover, salt stress activates plasma membrane RBOHs, contributing to the apoplastic ROS burst critical for initiating defense signaling (Mittler et al., 2004; Kurusu et al., 2015). The synergistic action of these mechanisms collectively amplifies the oxidative burst, making salt stress a distinct and complex form of oxidative stress.

#### 2.2.3 High temperature stress

High temperature stress impairs multiple developmental stages in plants. High temperature stress specifically damages PSII, leading to the degradation of D1/D2 proteins and reduced thylakoid membrane fluidity, and the mitochondrial electron transport chain, leading to excessive ROS accumulation in these organelles (Wahid et al., 2007; Hendrix et al., 2023). Apart from organellar sources, RBOHs act as crucial amplifiers of ROS signaling under high temperature (Drerup et al., 2013). For example, rice *OsRbohB* is strongly upregulated by heat across growth stages, with its activity finely regulated via calcium-mediated signaling and phosphorylation cascades (Liu et al., 2025). In maize, ZmCDPK7 enhances ROS accumulation by phosphorylating RBOHB under high temperatures (Zhao et al., 2021). In addition, protein misfolding induced by high temperature activates the unfolded protein response, further promoting ROS accumulation and forming a vicious cycle of “protein damage-ROS outburst”. This cycle is particularly detrimental during sensitive developmental stages. For instance, during reproductive growth in maize, heat stress can cause mitochondrial anomalies and endoplasmic reticulum dysfunction, leading to a sharp increase in ROS and decreased fertility. Thermotolerant cultivars, however, mitigate such damage through rapid induction of heat shock transcription factors (HSFs) and heat shock proteins (HSPs), thereby better maintaining redox homeostasis (Wang et al., 2025). Thus, under high-temperature stress, ROS serve not only as cytotoxic agents but also as key signaling molecules that mobilize defense mechanisms, critically shaping varietal heat tolerance.

#### 2.2.4 Low temperature stress

Low temperature stress (typically defined as 0-15 °C) significantly enhances ROS by impairing electron transport chains (Xiong et al., 2002). Chloroplasts exhibit particular sensitivity to chilling stress, as low temperatures not only reduce thylakoid membrane fluidity but also suppress the activity of key Calvin cycle enzymes (e.g., Rubisco), thereby disrupting the balance between light energy absorption and utilization and promoting ROS production (Gorsuch et al., 2010; Guan et al., 2023). Conversely, as an adaptive response, plants can maintain membrane fluidity by increasing ω-3 fatty acid content, which helps reduce electron leakage and ROS production at the source (Zhang et al., 2024a). In *Arabidopsis*, cold stress compromises the function of FtsH2 protease, a key regulator of PSII protein homeostasis. The impaired FtsH2 activity hinders PSII repair, exacerbating electron leakage in the thylakoid electron transport chain and promoting substantial ¹O_2_ production, which further induces oxidative damage (Zhang et al., 2024a). Mitochondria serve as another major ROS source: low temperature induces dysfunction of mitochondrial Complex I, promoting O_2_•^-^ release; in rice, the pentatricopeptide repeat protein SOP10, localized to mitochondria, directly modulates ROS production by regulating complex I function (Zu et al., 2023). This finding reveals a novel mechanism for fine-tuning ROS levels through modulation of specific mitochondrial electron transport chain components.

#### 2.2.5 Heavy metal stress

Heavy metals, such as cadmium (Cd), nickel (Ni), and chromium (Cr), are major environmental pollutants that disrupt ROS metabolism through persistent and cumulative effects. Heavy metals trigger ROS bursts through multiple, often interconnected, mechanisms. redox-active metal ions (e.g., Cu^+^/Cu²^+^, Cr³^+^/Cr^6+^) can directly participate in Fenton and Haber-Weiss reactions, catalyzing the formation of •OH and thereby markedly amplifying oxidative damage (Mithofer et al., 2004; Anjum et al., 2014). The specific molecular mechanisms of ROS induction vary with the chemical properties of the metal. For instance, Cd²^+^ a non-redox-active metal, indirectly promotes ROS accumulation by competitively displacing Ca²^+^ from proteins like calmodulin. In contrast, redox-active metals such as Cr^6+^ and Cu²^+^ directly catalyze •OH production via Fenton-type reactions (Pandey, 2018). Collectively, these processes induce extensive oxidative damage, ultimately severely impairing plant growth and development.

#### 2.2.6 UV-B stress

UV-B radiation (280–315 nm) inflicts direct damage to biomolecules such as DNA and protein structures, while indirectly impairing the photosynthetic ETC. These disruptions lead to ROS bursts and subsequent oxidative stress (Teramura and Sullivan, 1994; Frohnmeyer and Staiger, 2003). UV-B markedly reduces photosynthetic efficiency by damaging thylakoid membrane integrity and promoting the degradation of D1/D2 proteins in PSII, which impairs electron transfer at key donor-acceptor sites (e.g., QA, QB). Furthermore, UV-B suppresses Rubisco activity and inhibits the Calvin-Benson cycle, resulting in over-reduction of the ETC and promoting O_2_•^-^ and ¹O_2_ production (Kataria et al., 2014). The resulting ROS accumulation increases malondialdehyde (MDA) content and elevates membrane permeability (reflected by elevated relative electrical conductivity), thereby directly compromising membrane integrity (Joshi et al., 2011; Kataria et al., 2014). These physiological alterations manifest as reduced leaf size, diminished chlorophyll content, and lower stomatal conductance, collectively leading to photosynthetic inhibition.

#### 2.2.7 Ozone stress

Tropospheric O_3_ is a major photochemical air pollutant that severely threatens plant health and agricultural productivity. It primarily induces ROS production by infiltrating plant tissues through stomatal apertures, rapidly decomposing in the intercellular spaces and apoplast to generate ROS such as O_2_•^-^ and H_2_O_2_ (Kangasjärvi et al., 2005; Wrzaczek et al., 2009; Grulke and Heath, 2020). Guard cells exhibit a biphasic pattern of ROS accumulation, with chloroplasts acting as the primary ROS source, followed by ROS production in guard cell membranes, which required NADPH oxidases encoded by the *AtrbohD* and *AtrbohF* genes, thereby driving stomatal closure to reduce further O_3_ uptake (Joo et al., 2005; Vahisalu et al., 2010). Under persistent stress, ROS diffuse into mesophyll tissues, where chloroplasts and mitochondria become key sites for intracellular ROS amplification (Singh et al., 2014). However, under chronic or acute exposure, the spatial accumulation of ROS, particularly in mesophyll tissues, becomes critically linked to the activation of PCD (Overmyer et al., 2000). Histochemical studies in mung bean and clover have confirmed H_2_O_2_ localization following chronic O_3_ stress, correlating with visible leaf lesions (Chaudhary and Agrawal, 2013; 2015). In maize varieties exposed to ambient O_3_ concentrations elevated by +30 ppb, both H_2_O_2_ and O_2_•^-^ were detected in mesophyll tissues (Singh et al., 2014). Consequently, when ozone stress exceeds the capacity of the plant’s intrinsic defense mechanisms, it triggers irreversible oxidative damage.

The stress-specific mechanisms of ROS production outlined above, such as photoinhibition under drought and ionic toxicity under salinity, directly lead to various types of biomolecular damage. A comparative summary of these mechanisms across major abiotic stresses is provided in **Table 1**. To counter this damage and maintain cellular homeostasis, plants engage precisely regulated antioxidant networks.

### 2.3 The dual role of ROS: from signaling to oxidative damage

As in the previous sections, different abiotic stresses modulate the magnitude and spatiotemporal dynamics of ROS production in a highly specific manner. The ultimate biological outcome of these ROS, whether they act as crucial signaling molecules or as agents of cellular destruction, is critically determined by their concentration, duration, and site of accumulation (Mittler, 2002; Miller et al., 2010). This concentration-dependent functional duality positions ROS as a central “double-edged sword” in plant stress biology. This section establishes a conceptual framework for understanding this duality, thereby setting the stage for the subsequent discussion on its precise regulation by plants.

#### 2.3.1 ROS as central signaling hubs under abiotic stress

At low physiological concentrations, ROS act as essential second messengers that regulate a wide array of physiological and developmental processes. Their signaling capacity is characterized by precision, adaptability, and systemic reach, forming a dynamic communication network that enables plants to perceive and respond to environmental cues. For instance, H_2_O_2_ serves as a key regulator of stomatal movement by modulating ion channel activities in guard cells, thereby mediating plant adaptation to drought and other osmotic stresses (Sharova et al., 2023). Beyond immediate stress responses, ROS are also integral to regulating PCD, playing critical roles in developmental processes such as xylem differentiation and in the elimination of damaged cells under various stress conditions (Baxter et al., 2014; Wang et al., 2023a). In summary, ROS serve as a dynamic, self-regulating signaling hub in plant stress responses. However, under sustained or intense stress, these regulatory capacities can be overwhelmed, converting precise ROS signals into triggers of widespread oxidative damage.

The signaling potential of ROS is highly context-dependent. The same ROS molecule can trigger divergent physiological fates depending on the cellular environment. For instance, a localized ROS burst can induce PCD in specific tissues (e.g., the tapetum in rice anthers under heat stress), while in other cells, chloroplast-derived H_2_O_2_ can act as a retrograde signal to activate transcription factors that enhance thermotolerance (Ling et al., 2025). Specifically, ROS signals are decoded into diverse physiological outputs: under salt stress, the RBOH-mediated ROS burst directly contributes to ion homeostasis by modulating transporters such as SOS1, forming a regulatory loop that fine-tunes Na^+^/K^+^ balance (Ma et al., 2012; Jiang et al., 2012; Wu et al., 2024); under heavy metal stress, they induce detoxification pathways (Pandey, 2018); and under cold stress, they modulate lipid desaturation to reduce ROS production at the source (Zhang et al., 2024a).

Furthermore, ROS signals can propagate systemically, transforming local perception into whole-plant acclimation. This is exemplified by RBOH-mediated “ROS waves” that communicate stress signals from roots to shoots, coordinating avoidance responses and remodeling growth architecture (Wang et al., 2023b). In summary, at controlled levels, ROS constitute a precise, adaptable, and systemic signaling hub essential for stress perception and integration.

#### 2.3.2 ROS-induced oxidative damage: mechanisms and consequences

When ROS production persistently exceeds the cellular scavenging capacity, the homeostatic equilibrium is disrupted, leading to their excessive accumulation and the onset of oxidative stress (Mittler, 2002). At elevated concentrations, ROS non-selectively oxidize vital cellular components—including proteins, lipids, and nucleic acids—causing structural damage, metabolic dysfunction, and ultimately growth inhibition or even plant mortality (Choudhury et al., 2017).

Among these detrimental effects, lipid peroxidation represents a major outcome of oxidative stress. Highly reactive ROS (e.g., •OH and ¹O_2_) initiate lipid peroxidation by attacking the double bonds of unsaturated fatty acids in membrane lipids. This triggers radical chain reactions, generating lipid hydroperoxides as primary products. These unstable compounds subsequently decompose into cytotoxic aldehydes, notably MDA. The resulting alteration in lipid composition irreversibly increases membrane fluidity and permeability, disrupting cellular compartmentalization and leading to ion leakage and metabolic dysfunction (Dhindsa et al., 1982; Wang et al., 2023). Furthermore, MDA can form cross-links with amino groups of proteins and bases of DNA, generating stable MDA-biomolecule adducts that further amplify oxidative damage (Choudhury et al., 2017). Consequently, MDA content is widely employed as a key biomarker for assessing the severity of oxidative stress in plants.

Proteins represent another crucial target of ROS assault. Their structure and function can be impaired through direct and indirect modification mechanisms. Direct modifications include carbonylation (the introduction of carbonyl groups via •OH-mediated oxidation of amino acid side chains), disulfide bond disruption (through H_2_O_2_-induced oxidation of thiol groups), and glutathionylation (the covalent attachment of glutathione to proteins). Together, these modifications lead to enzyme inactivation, protein misfolding, and loss of functional three-dimensional structure (Choudhury et al., 2017). Indirect modifications mainly involve the formation of adducts between lipid peroxidation products (e.g., MDA) and protein amino groups, which induce intra- and inter-polypeptide cross-linking and generate insoluble aggregates. This process disrupts the dynamic balance between protein degradation and synthesis (Mittler et al., 2011; Schieber and Chandel, 2014; Choudhury et al., 2017). The physiological repercussions of these modifications inactivate enzymatic active sites, promote protein misfolding and aggregation, and consequently disrupt cellular signaling and metabolic homeostasis (Schieber and Chandel, 2014; Choudhury et al., 2017). Notably, Critical enzymes involved in photosynthesis (e.g., Rubisco) and respiration are particularly susceptible, directly compromising the plant’s energy metabolism. Therefore, protein damage by ROS significantly impairs plant vitality and overall physiological function.

In addition to damaging membranes and proteins, ROS inflict substantial damage on genetic material. Nuclear, chloroplast, and mitochondrial DNA are all vulnerable to ROS attack, sustaining damage including deoxyribose oxidation, strand breaks, base modifications, and DNA-protein cross-links (Schieber and Chandel, 2014; Choudhury et al., 2017). A particularly characteristic DNA oxidation product is 8-hydroxy-2’-deoxyguanosine (8-OHdG), which results from •OH-mediated abstraction of a hydrogen atom from the C-8 position of guanine (Mittler, 2002; Choudhury et al., 2017). Unless efficiently repaired, such damage represents a fundamental source of gene mutations and cellular dysfunction, posing a serious threat to plant genetic integrity and normal growth and development.

#### 2.3.3 The dynamic threshold: integrating signaling and damage

ROS play a quintessential dual role in plant abiotic stress responses: they act as indispensable signaling messengers under homeostatic conditions, yet turn into potent cytotoxic agents when redox balance is disrupted (Mittler, 2002; Wang et al., 2023). The boundary between these two opposing functions is defined by a “dynamic threshold”, which is not fixed but shaped by the interplay of stress type, intensity, duration, and the plant’s intrinsic antioxidant capacity (Mittler et al., 2022; Myers et al., 2024). At controlled levels, localized in specific subcellular compartments and sustained for short periods, ROS mediate key adaptive responses such as stomatal movement, PCD, and systemic acclimation via “ROS waves” (Myers et al., 2024). However, when stress persists or intensifies, ROS production exceeds scavenging capacity, leading to uncontrolled accumulation that oxidizes lipids, proteins, and nucleic acids, ultimately inhibiting growth or inducing cell death.

This dynamic threshold serves as the core target of plant redox regulation, with plants strategically adjusting their responses to navigate ROS “double-edged sword” nature across diverse stresses (Wang et al., 2023; Myers et al., 2024). For instance, drought and salt stress prioritize retaining ROS as transient signals to trigger osmotic and ion balance adjustments, while heavy metal stress focuses on rapid ROS clearance to mitigate the toxicity of hydroxyl radicals generated via Fenton reactions. This stress-specific calibration of the threshold underscores the central paradox of ROS in plant stress biology, their necessity for signaling and potential for destruction (Mittler et al., 2022). The following sections will elaborate on the sophisticated regulatory networks that fine-tune this threshold, enabling plants to maintain ROS homeostasis and achieve stress acclimation.

#### 2.3.4 ROS waves as systemic manifestations of redox signaling

ROS waves are essential for the induction of key systemic acclimation processes, including systemic acquired acclimation (SAA) to abiotic stress, the systemic wound response (SWR), and SAR to pathogens, thereby ensuring coordinated survival responses across the plant (Myers et al., 2024). This ensures coordinated survival strategies across the entire plant, extending the dual-role paradigm of ROS from cellular to whole-plant scales. Addressing the core question of conservation versus stress-specificity, both the propagation mechanisms and physiological functions of ROS waves exhibit a pattern of conserved core components modulated by stress-specific regulatory cues.

ROS wave propagation primarily relies on the following mechanisms: (i) A relay mechanism driven by sequential activation of RBOH proteins (e.g., *Arabidopsis* RBOHD/F, rice OsRbohB), which are synergistically activated by Ca²^+^ binding to EF-hand motifs and phosphorylation by CDPKs or CBL-CIPK modules, generating a self-propagating apoplastic ROS front via the “Ca²^+^ influx—ROS production—more Ca²^+^ influx” positive feedback loop (Miller et al., 2009; Drerup et al., 2013); (ii) Facilitation via plasmodesmata (PD) for local cell-to-cell movement, where RBOHD-derived apoplastic ROS upregulates PD-localized proteins (PDLP1/5) to expand PD aperture, amplifying signal transmission and participating in local and systemic acclimation (Peláez-Vico et al., 2023); (iii) Integration into a rapid “ROS-Ca²^+^-hydraulic-electric” systemic signaling network for long-distance transmission. Ca²^+^-permeable channels (GLR3.3/3.6, MSL3) mediate Ca²^+^ wave synchronization; electric waves depend on GLR3.3/3.6-mediated membrane depolarization; hydraulic waves release L-glutamate to activate systemic signals; and plant hormones (ABA, SA, JA) and damage-associated molecular patterns (DAMPs, e.g., eATP, L-glutamate) fine-tune network activity (Ali and Muday, 2024; Myers et al., 2024).

Functionally, ROS waves directly manifest the concentration-dependent logic of the ROS dynamic threshold. They serve a conserved dual purpose: as a rapid systemic alert (e.g., underpinning SWR or inducing stomatal closure within minutes during ozone stress) and as an orchestrator of long-term systemic acclimation (e.g., SAA to abiotic stress or SAR to biotic stress) (Mittler et al., 2022; Myers et al., 2024). The dominant output is stress-tailored: acute threats prioritize rapid alert, whereas chronic stresses emphasize sustained acclimation, illustrating how a conserved propagation module is decoded into context-appropriate whole-plant responses.

## 3 ROS scavenging under abiotic stresses​

To mitigate oxidative damage caused by excessive ROS accumulation under stress conditions, plants have evolved a sophisticated, multi-layered antioxidant defense network to maintain intracellular redox homeostasis. This network comprises enzymatic and non-enzymatic systems working synergistically.

### 3.1 The antioxidative defense system in plants

#### 3.1.1 Enzymatic antioxidants in ROS scavenging

The enzymatic antioxidant system consists of a suite of core enzymes that catalyze the detoxification and elimination of intracellular ROS.

SOD acts as the first line of defense against ROS, dismutating O_2_•^-^ to H_2_O_2_ and O_2_. Its isoforms (Cu/Zn-, Mn-, Fe-SOD) show precise subcellular localization (e.g., cytosol, chloroplasts, mitochondria, apoplast), ensuring immediate interception of O_2_•^-^ near its site of generation ensuring immediate interception of O_2_•^-^ near its site of generation (Mittler, 2002; Pilon et al., 2011). SOD activity is frequently utilized as a key physiological indicator of a plant’s intrinsic stress tolerance.

CAT, predominantly localized in peroxisomes, detoxifies high concentrations of H_2_O_2_ by catalyzing its direct decomposition into H_2_O and O_2_ (Mittler et al., 2011). It plays a major role in scavenging H_2_O_2_ produced during fatty acid β-oxidation and photorespiration, thereby preventing its diffusion into the cytosol and minimizing oxidative damage (Gechev et al., 2006; Wang et al., 2023a). Under stress conditions, CAT activity is often significantly enhanced, which is of great importance for preserving the integrity of membrane structures.

APX are central to H_2_O_2_ scavenging in chloroplasts and the cytosol and utilizes AsA as an electron donor t donor with high affinity for H_2_O_2_, generating monodehydroascorbate (MDHA) as an intermediate (Gill and Tuteja, 2010). APX exists as multiple isoforms distributed across various subcellular compartments, such as chloroplasts, the cytosol, mitochondria, and peroxisomes, enabling precise and efficient H_2_O_2_ clearance at its distinct sites of generation (Gill and Tuteja, 2010; Anjum et al., 2014).

Glutathione reductase (GR) plays a central role in maintaining the cellular reduced GSH pool by catalyzing the NADPH-dependent reduction of oxidized GSSG to GSH, thereby upholding the crucial GSH/GSSG redox balance (Anjum et al., 2014). This recycling is indispensable for the proper functioning of ascorbate-glutathione (AsA-GSH) cycle and other GSH-dependent detoxification pathways.

#### 3.1.2 Non-enzymatic antioxidant components

Beyond the precisely regulated enzymatic machinery, plants employ a diverse array of low-molecular-weight metabolites that collectively constitute a rapid-response, metabolite-driven antioxidant layer.

As the most abundant water-soluble antioxidants in plant cells, AsA and GSH are core redox buffers. AsA, the primary electron donor for APX in the AsA-GSH cycle, directly scavenges multiple ROS species and helps regulate photosynthetic electron flow to minimize ROS generation at its source (Noctor and Foyer, 1998). GSH, beyond being a substrate for detoxification enzymes (e.g., GPX, GST), critically modulates protein function via glutathionylation, with the GSH/GSSG ratio serving as a master indicator of cellular redox poise (Noctor and Foyer, 1998; Anjum et al., 2014).

Beyond AsA and GSH, plants employ a suite of other non-enzymatic antioxidants that fulfill critical protective roles. Phenolic compounds (e.g., flavonoids, anthocyanins) neutralize free radicals and terminate lipid peroxidation chains through hydrogen atom donation (Mittler et al., 2004; Gill and Tuteja, 2010). Lipophilic antioxidants., such as carotenoids and vitamin E (α-tocopherol), are embedded in photosynthetic membranes, in which carotenoids quench singlet oxygen and triplet chlorophyll, while vitamin E halts lipid peroxidation by trapping peroxyl radicals, collectively preserving membrane integrity (Mittler et al., 2004; Gill and Tuteja, 2010). Under stress, plants accumulate compatible solutes such as proline and glycine betaine. In addition to osmotic adjustment, these solutes contribute to oxidative stress mitigation by acting as direct ROS scavengers and chemical chaperones that stabilize proteins and membranes, thereby supporting overall cellular homeostasis (Mittler et al., 2004; Gill and Tuteja, 2010; Rasheed et al., 2024).

In summary, the non-enzymatic system provides immediate, versatile chemical defense and robust redox buffering. Its synergistic interplay with the precisely targeted enzymatic system creates a resilient, multi-tiered network, enabling plants to dynamically manage oxidative stress and balance metabolic investment between growth and defense in fluctuating environments.

### 3.2 Regulation of ROS homeostasis under abiotic stress conditions​

**Figure 1:**
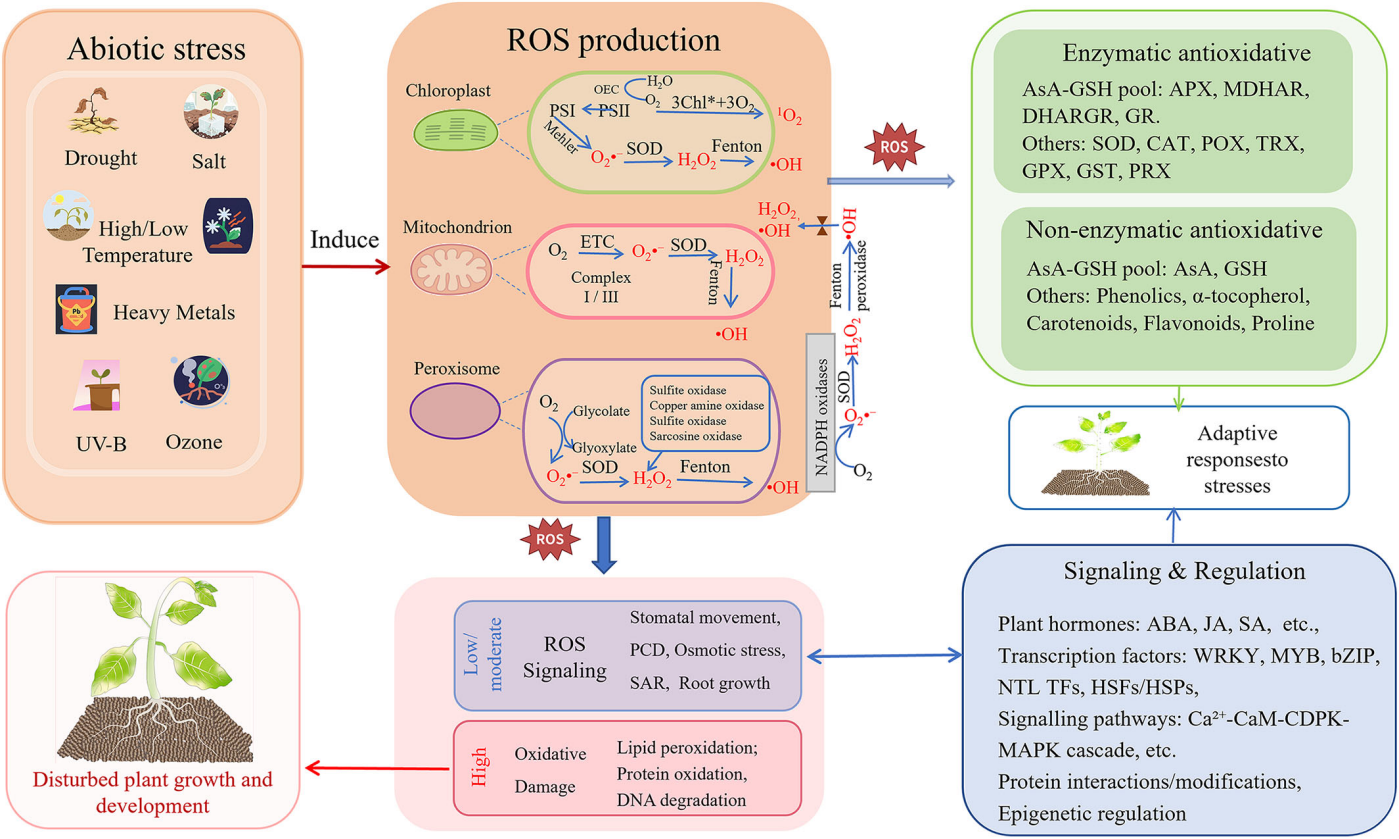
Production, scavenging, and signaling network of reactive oxygen species (ROS) in plants under abiotic stresses. This model summarizes the integrated response of plants to abiotic stresses (e.g., drought, salinity, extreme temperatures, heavy metals, UV-B, ozone). Abiotic stressors induce ROS overproduction mainly in chloroplasts, mitochondria, peroxisomes, and plasma membrane via disrupted electron transport or metabolic imbalances. ROS act as a “double-edged sword”: at physiological levels, they act as signaling molecules mediating adaptive responses (e.g., stomatal movement, PCD, SAR, root growth); excessive accumulation leads to oxidative damage (e.g., to lipids, proteins, DNA). To maintain redox balance, plants employ synergistic antioxidant systems, including enzymatic (e.g., SOD, CAT, AsA-GSH cycle) and non-enzymatic (e.g., AsA, GSH, flavonoids) components. This integrated network is dynamically regulated by phytohormones (e.g., ABA, JA, SA), transcription factors (e.g., WRKY, MYB, HSFs), Ca²^+^-CaM-CDPK-MAPK signaling cascades, post-translational modifications, and epigenetic mechanisms, culminating in an adaptive stress response.

Although plants possess a conserved antioxidant defense system, they implement differential reprogramming under different stresses through transcriptional regulation, post-translational modifications, and hormonal crosstalk, as summarized in **Figure 1** and **Table 1**, thereby achieving optimal ROS scavenging efficiency.

#### 3.2.1 Drought stress

To mitigate drought-induced oxidative damage, plants activate a multi-layered, highly coordinated antioxidant defense and regulatory network. Under drought stress, the activities of SOD, CAT, and POD are often significantly enhanced to cope with increased ROS levels (Selote et al., 2004; Yao et al., 2025). In wheat (*Triticum aestivum* L.), *TaWD40-4B.1C* interacts with TaCAT, promoting its oligomerization and enhancing enzymatic activity, thereby improving H_2_O_2_ scavenging capacity. Notably, TaCAT deficiency completely abolishes *TaWD40-4B.1C*-mediated drought tolerance (Tian et al., 2023). In *Arabidopsis*, the calcium-dependent protein kinase CPK8 regulates CAT3 activity and subcellular localization through phosphorylation, while the E3 ubiquitin ligase CIRP1 mediates ubiquitination-dependent degradation of CAT2 and CAT3, negatively regulating drought tolerance and oxidative stress resistance (Yang et al., 2025; Zou et al., 2015).

The regulation of ROS homeostasis under drought also relies on hormonal signaling and post-translational modifications. Phytohormones play a central role in integrating drought signals with antioxidant defense. ABA, as a core drought responses regulator, promotes YUCCA-mediated auxin (IAA) biosynthesis in sorghum seedlings under drought stress. This coordinated response enhances activities of antioxidant enzymes including SOD and POD, synergistically reducing O_2_•^-^ and H_2_O_2_ levels while alleviating membrane lipid peroxidation (Yao et al., 2025). Other phytohormones such as Brassinosteroids (BRs), along with regulatory genes, also contribute to drought resistance by modulating this antioxidant system (You and Chan, 2015). At the protein level, kinase-mediated phosphorylation (e.g., by CPK8) and E3 ligase-mediated degradation (e.g., by CIRP1) dynamically regulate the abundance and activity of antioxidant enzymes like CAT (Yang et al., 2025; Zou et al., 2015). Furthermore, key RBOH isoforms, such as *Arabidopsis RbohD*, demonstrates dual functionality as both a ROS producing enzyme and a selective autophagy cargo receptor. This creates a feedback loop where RbohD-derived ROS can initiate autophagy, which in turn, through RbohD itself, facilitates the turnover of ROS-producing complexes, thereby dynamically modulating signal output and maintaining homeostasis (Chen et al., 2025). Such synergistic regulation between hormones signaling and antioxidant system enhances plant adaptability to varying drought intensities.

#### 3.2.2 Salt stress

Salt stress induces the upregulation of various antioxidant enzymes to mitigate oxidative damage (Hernandez et al., 2001). Recent studies have progressively elucidated the roles of specific regulatory factors in maintaining ROS homeostasis under salt stress (Liu et al., 2021). In *Arabidopsis*, LBD11 maintains O_2_**·**^-^ homeostasis within the root apical meristem by coordinately regulating the peroxidase gene *PRX7* and the NADPH oxidase genes *RBOHD*/*RBOHF* (Dang et al., 2025). In sand willow (*Salix psammophila*), the SpsWRKY53–SpsMDP1–SpsPP2C80 module amplifies the ROS cycle by enhancing SOD and POD activities in coordination with ABA signaling, establishing a ROS-ABA positive feedback loop (Li et al., 2025). In rice, OsCPK12 enhances the catalytic activity of catalases OsCATA and OsCATC through phosphorylation of Ser11 residues, effectively regulating H_2_O_2_ homeostasis and significantly improving oxidative stress tolerance (Wang et al., 2024). In wheat, TaWRKY44 enhances ROS scavenging capacity by inducing expression of the dehydrin gene *TaDHN7* (Jia et al., 2025). Similarly, in *Brassica napus*, BnBBX22.A7 mitigates oxidative damage and enhances salt tolerance by activating components of the ROS scavenging system (Zhang et al., 2024b). Moreover, in soybean under salt stress, the oxidized transcription factor GmNTL1 translocate to the nucleus to directly activate *GmRbohB* genes, amplifying the ROS signal in a feed-forward manner (Zhang et al., 2023b). Beyond local regulatory circuits, systemic ROS signaling plays a crucial role in whole-plant acclimation. Under asymmetric salt stress, for instance, rice roots employ OsRBOHA and OsRBOHI to generate a systemic ROS wave that coordinates localized avoidance responses across tissues (Wang et al., 2023b). Collectively, these studies demonstrate that plants employ complex regulatory networks involving transcription factors, protein modules, and signaling pathways to precisely coordinate ROS production, signaling, and scavenging, thereby achieving synergistic enhancement of salt stress adaptation.

Halophytes have evolved sophisticated and multifaceted mechanisms for regulating ROS homeostasis, which differ fundamentally from glycophytes. One key aspect of halophyte adaptation lies in their superior compartmentalization through the expansion of key gene families and reduced ROS production at the source. For instance, genomic analyses reveal that *Suaeda salsa* possesses a significantly expanded vacuolar membrane Na^+^/H^+^ antiporter (NHX) gene family (Cui et al., 2025), which confers enhanced Na^+^ sequestration capacity. This efficient compartmentalization of excess Na^+^ into vacuoles thereby reduces ROS generation originating from chloroplasts and mitochondria (Bose et al., 2014; Song and Wang, 2015). Integrative physiological and multi-omics analysis further reveals that the salt stress response in *S. salsa* is characterized by gradient responses, functional differentiation, and energy optimization, with leaves playing a dominant role in systemically coordinating ion balance and antioxidant defense, thereby optimizing the control of ROS production at its source (Tang et al., 2025). In addition to such ion compartmentalization strategies, some halophytes may employ other physiological adaptations, such as photosynthetic mode transitions (e.g., from C3 to CAM or C4 pathways), to minimize ROS production (Sunagawa et al., 2010; Uzilday et al., 2012).

Furthermore, halophytes exhibit an enhanced and finely tuned antioxidant defense system. This enhanced capacity is not merely due to higher expression of common enzymes but is fundamentally rooted in evolutionary innovations such as the expansion and neofunctionalization of key gene families. For example, halophytes such as *Salicornia europaea* demonstrate significantly greater upregulation of SOD, APX, and CAT activities and corresponding gene expression under salt stress compared to glycophytes like *Arabidopsis* (Bose et al., 2014; Song and Wang, 2015; Kumar et al., 2021). This efficient regulation is exemplified in the halophyte *Cakile maritima*, where H_2_O_2_ accumulation peaks within 4 hours following 400 mM NaCl treatment and subsequently declines rapidly, whereas in *Arabidopsis*, H_2_O_2_ continues to accumulate for up to 72 hours even under milder salt stress (100 mM NaCl) (Ellouzi et al., 2011). This superior antioxidant performance is underpinned by key evolutionary innovations, including the expansion of gene families and coordinated metabolic reprogramming. In *Suaeda salsa*, comparative genomic analysis reveals significant expansion of gene families related to flavonoid biosynthesis, phenylpropanoid metabolism, and trace element (e.g., Zn, Fe, Mn) binding proteins (Cui et al., 2025). These trace elements serve as essential cofactors for antioxidant enzymes such as SOD. Integrated transcriptomic and metabolomic data further confirm that under high salinity, pathways including phenylpropanoid biosynthesis and starch and sucrose metabolism are specifically activated in *S. salsa*, promoting the accumulation of antioxidant metabolites such as flavonoids (Tang et al., 2025; Cui et al., 2025). These metabolites act synergistically with the enzymatic antioxidant system to form an efficient and robust ROS-scavenging network. This multilevel strategy—combining enhanced enzymatic regulation, metabolite-driven defense, and metabolic optimization—represents a key evolutionary adaptation that distinguishes halophytes from glycophytes in coping with oxidative stress.

Halophytes actively coordinate ROS homeostasis with broader stress responses through evolutionarily refined regulatory networks, rather than merely scavenging ROS passively. In *S. salsa*, salt stress responses are orchestrated in a leaf-dominant manner, with transcriptomic profiling revealing pronounced upregulation of genes involved in hormone signaling (e.g., ABA, JA), MAPK pathways, and secondary metabolite biosynthesis—responses that are more robust in salt-tolerant *S. salsa* than in *S. glauca* (Tang et al., 2025; Yan et al., 2024). This enables halophytes to integrate ROS signaling with ion homeostasis, osmotic adjustment, and antioxidant defense, thereby maintaining ROS at a controlled, functionally beneficial signaling level. Notably, halophytes actively utilize ROS as signaling molecules, for example, in *Salicornia herbacea*, salicylic acid synergistically regulates ion and ROS homeostasis through activation of RbohF-mediated H_2_O_2_ signaling (Wu et al., 2024). Thus, beyond efficient ROS elimination, halophytes leverage evolved regulatory networks to harness ROS as integral signals within a coordinated stress−acclimation system.

#### 3.2.3 High temperature stress

To combat heat-induced oxidative injury, plants deploy multi-layered antioxidant systems. Under heat stress, the activities of key antioxidant enzymes are dynamically modulated. For instance, in red raspberry, CAT activity increases progressively under 35 °C and 40 °C stress, whereas SOD activity rises initially but declines thereafter, illustrating phase-specific coordination of scavenging enzymes (Guo et al., 2023). At the molecular level, post-translational modifications precisely regulate enzyme function: in tomato, CPK28 interacts with and stabilizes APX2, thereby augmenting thermotolerance (Hu et al., 2021). Concurrently, transcriptional regulation fine-tunes defense, in tall fescue, the transcription factor FaNAC047 promotes chlorophyll degradation and ROS accumulation by directly activating chlorophyll degradation genes (*FaNYC1*, *FaNOL*, *FaSGR*) while repressing the antioxidant gene *FaCAT2*, thereby accelerating heat-induced leaf senescence (Cao et al., 2025).

The regulation of ROS homeostasis under heat stress is deeply integrated with hormonal signaling and programmed signal-decoding networks. In rice anthers, the ABA-SAPK2-RH4 pathway regulates ROS homeostasis under high temperatures, excessive ROS induces PCD in the tapetum, leading to pollen abortion (Zhao et al., 2023a). Conversely, in vegetative tissues, chloroplast-derived H_2_O_2_ in other tissues can act as a retrograde signal to activate the heat shock factor HSFA2-2, establishing a reinforcing feedback loop (“EGY3–H_2_O_2_–HSFA2-2” that enhances thermotolerance (Ling et al., 2025). Beyond classical hormones, volatile isoprenoids (e.g., isoprene and monoterpenes) not only scavenge ROS directly but also function as signaling molecules that activate the have been shown to function as signaling molecules that activate the Ca²^+^-CDPK-MAPK cascade, subsequently inducing HSPs and antioxidant enzymes (Zuo et al., 2025). In summary, plants dynamically maintain ROS homeostasis under high-temperature stress by integrating enzymatic regulation, transcriptional control, and multiple signaling pathways. This coordinated response enables a critical balance between acclimation and survival, revealing new targets for breeding thermotolerant crops.

#### 3.2.4 Low temperature stress

Under low temperatures, the activities of key antioxidant enzymes including SOD, APX, DHAR, MDHAR, and GR are generally enhanced (Gorsuch et al., 2010). This enzymatic defense is complemented by the accumulation of non-enzymatic antioxidants. In apple, MdbHLH33, MdMYB308L, MdMYB23, and MdMYBPA1 promote anthocyanin and proanthocyanidin accumulation by directly binding to promoters of flavonoid biosynthesis genes, thereby enhancing ROS scavenging capacity and membrane stability (An et al., 2020). In kiwifruit, the cold-induced bZIP transcription factor AcePosF21 interacts with AceMYB102 to directly bind the promoter of GDP-L-galactose phosphorylase 3 (*AceGGP3*), a key gene in AsA biosynthesis, thereby promoting AsA production. This process facilitates the elimination of excess ROS generated by chilling injury and enhances cold tolerance (Liu et al., 2023).

The regulation of ROS homeostasis under low temperature is deeply integrated with transcriptional control and signaling networks. Specific transcription factors form central hubs in this response.in pummelo (*Citrus grandis*), PtrbHLH regulates ROS homeostasis by activating *PtrCAT* expression (Geng et al., 2019). Beyond transcription, epigenetic and post-translational mechanisms provide additional layers of precise regulation. In *Chrysanthemum*, DgMYB and DgATX form a regulatory module that activates the peroxisomal gene *DgPOD* expression via histone H3K4me3 modification (Luo et al., 2024). At the protein level, direct modulation of ROS-producing complexes represents a key regulatory strategy. In rice, the mitochondrial-localized pentatricopeptide repeat protein SOP10 fine-tunes ROS production by regulating complex I function. This modulation reduces O_2_^.-^ accumulation under low temperature, effectively alleviating oxidative leaf bleaching and enhancing cold tolerance (Zu et al., 2023). In summary, Plants form a multi-level defense network by enhancing antioxidant systems and stabilizing membranes to counteract low-temperature oxidative stress.

#### 3.2.5 Heavy metal stress

To mitigate heavy metal-induced oxidative stress, the chromium hyperaccumulator *Leersia hexandra* significantly enhances the activities of antioxidant enzymes (APX, SOD, CAT) and glutathione levels under combined chromium-nickel stress (Fu et al., 2024). In rye grass exposed to copper or lead stress, leaves exhibit significant adaptive changes in antioxidant enzyme activities (SOD, CAT, POD), total antioxidant capacity, and levels of biomarkers like metallothionein (Ran et al., 2025). Non-enzymatic metabolites reinforce ROS scavenging and metal detoxification. *Leersia hexandra* activates secondary metabolic pathways involving flavonoids and glucosinolates for synergistic detoxification under combined chromium-nickel stress (Fu et al., 2024). Plants often accumulate compatible solutes (e.g., proline, glycine betaine) and phenolic compounds under heavy metal stress, which contribute to ROS scavenging and detoxification. Crucially, the biosynthesis of phytochelatins and metallothioneins for metal chelation and vacuolar sequestration represents a frontline defense that reduces the availability of free metal ions for ROS-generating reactions (Gill and Tuteja, 2010).

Transcriptional and post-translational regulation fine-tunes antioxidant defenses. Prolonged Ni stress triggers the upregulation of transcription factors such as APX7 and SODCP, boosting the plant’s antioxidant capacity (Chen et al., 2024). Systemic signaling pathways, including hormone cascades (e.g., the RBOH-ROS-auxin cascade), reshape root architecture to avoid metal uptake and reduce ROS production at the source (Wang et al., 2023b). Thus, the integrated antioxidant and metal detoxification defenses, regulated at transcriptional and systemic levels, enable plants to maintain ROS homeostasis and underpin phytoremediation approaches.

#### 3.2.6 UV-B stress

Under prolonged UV-B exposure, elevated H_2_O_2_ levels have been observed in soybeans, rice, wheat, and other crops, concomitant with the activation of antioxidant enzymes such as SOD, POD, and CAT activities (Ma et al., 2019; Wang et al., 2023; Kirova et al., 2024). In wheat, for example, UV-B stress enhances the transcriptional activity of genes encoding key antioxidant enzymes, POX, GST, CAT, and SOD promoting ROS clearance (Kirova et al., 2024). Concurrently, non-enzymatic antioxidants, notably flavonoids and phenolic compounds, accumulate significantly in species such as soybean, sainfoin (*Onobrychis viciifolia* Scop.), and capiri wood (*Sideroxylon capiri* Pittier.), contributing to enhanced total antioxidant capacity and alleviation of oxidative injury (Yildirim, 2020; Martinez-Silvestre et al., 2022).

The regulation of ROS homeostasis under UV-B stress is deeply integrated with signaling pathways and transcriptional control. The UVR8 signaling pathway serves as a central hub: upon UV-B perception, UVR8 translocates to the nucleus and interacts with transcription factors such as HY5 to activate the expression of antioxidant enzyme genes and flavonoid biosynthesis genes (e.g., CHS, F3H) (Kataria et al., 2014). Together, the multi-layered defense mechanism collectively maintains cellular ROS homeostasis, representing a core strategy for plant adaptation to UV-B stress.

#### 3.2.7 Ozone stress

To counteract the phytotoxic oxidative burst triggered by ground-level ozone (O_3_), plants deploy a multi-layered defense network integrating enzymatic and non-enzymatic antioxidants, supported by sophisticated transcriptional and post-transcriptional regulation. However, their intrinsic capacity to neutralize O_3_-derived ROS is often limited, and sustained or acute exposure can readily overwhelm these constitutive defenses (Hasanuzzaman and Fujita, 2022). Enzymatic and non-enzymatic antioxidant systems act synergistically to scavenge excess ROS. For instance, in soybean leaves, O_3_ stress leads to decreased levels of AsA and GSH, alongside increased levels of DHA and GSSG. Concurrently, the activities of antioxidant enzymes such as APX, MDHAR, and GR initially increase but subsequently decline (Wang et al., 2011). Similarly, in grapevines, O_3_ stress induces ROS accumulation, and dysfunction of antioxidant-related proteins exacerbates ROS induced photodamage, highlighting the dependence on intact antioxidant machinery (Chen et al., 2020).

At the transcriptional level, O_3_ rapidly reprograms gene expression through microRNA-mediated mechanisms. In *Arabidopsis*, 22 miRNA families display altered expression within one hour of O_3_ exposure, reallocating cellular resources toward defense by repressing growth-related genes (Iyer et al., 2012). Thus, coordinated antioxidant and miRNA-mediated regulatory networks maintain ROS homeostasis under O_3_ stress, providing a basis for developing resilient crops.

In summary, plants maintain dynamic ROS homeostasis under various abiotic stresses through a sophisticated regulatory network involving precise control of ROS production, coordinated antioxidant activation, and intricate ROS signaling. While common mechanisms exist, each stress induces specific triggers and unique regulatory pathways, as compared in **Table 1**. Future research integrating multi-omics technologies, advanced imaging techniques, and gene editing tools to dissect these stress-specific ROS metabolic pathways and signaling cascades holds considerable promise for developing innovative strategies in breeding stress-tolerant crops and ensuring global food security in a changing climate.

## 4 Advances in ROS detection technologies

Accurate monitoring of the spatiotemporal dynamics of ROS—from their production to scavenging and signaling—is fundamental to understanding the processes described in previous sections. However, the high reactivity and short half-lives of ROS pose significant detection challenges. Recent advances have spurred innovative methods, substantially enhancing our capacity to analyze ROS *in vivo*. **Table 2** provides a comparative overview of these techniques.

**Table 2:** Comparison of major techniques for detecting ROS in plants.

Technology category	Technology type	Target ROS species	Sensitivity	Spatial & temporal resolution	Advantages	Limitations	Representative references
Traditional Histochemistry	DAB staining	H_2_O_2_	Low	Low (Tissue level, Static)	Inexpensive, simple, *in-situ* localization.	Qualitative/semi-quantitative, specific to H_2_O_2_, subjective, no dynamics.	(Jambunathan, 2010)
	NBT staining	O_2_•^-^	Low	Low (Tissue level, Static)	Inexpensive, simple, *in-situ* localization.	Qualitative, specific to O_2_•^-^, subjective, no dynamics.	(Jambunathan, 2010)
Chemical Fluorescent Probes	H_2_DCFDA	Broad-spectrum (H_2_O_2_, •OH)	High	High (Cellular, Quasi-dynamic)	Highly sensitive, suitable for confocal microscopy and flow cytometry.	Lacks specificity, prone to photo-oxidation and artifacts.	(Ortega-Villasante et al., 2016, 2018)
	NIR Probes (e.g., Cy-Bo)	H_2_O_2_	Very High (nM range)	High (Tissue depth, Quasi-dynamic)	Deep tissue penetration, low background, high sensitivity.	Requires specialized NIR imaging systems.	(Jin et al., 2025; Zhao et al., 2023b)
Genetically Encoded Sensors	HyPer	H_2_O_2_	High	High (Subcellular, Real-time)	Subcellular targeting, real-time monitoring in living plants.	pH-sensitive, requires genetic transformation.	(Lara-Rojas et al., 2023)
	roGFP2-Orp1	H_2_O_2_	High	High (Subcellular, Real-time)	pH-insensitive, real-time redox monitoring.	Requires genetic transformation.	(Nietzel et al., 2019; Denjalli et al., 2024)
Nanosensors	AIENPs@Mo/Cu-POM	H_2_O_2_	Very High (sub-µM)	High (Tissue, Multiplexed)	High sensitivity, multiplexing capability, potential for stress-type discrimination via machine learning.	Complex synthesis, nascent technology, not yet widely adopted.	(Hu et al., 2025)
	(GT)_15_-DNA-SWNT	H_2_O_2_, Ca²^+^, others	High	High (Tissue level, Real-time)	Multi-channel sensing, can monitor ROS wave propagation kinetics.	Complex data interpretation, nascent technology.	(Ang et al., 2024; Bao et al., 2024)

### 4.1 Traditional histochemical methods

Traditional ROS detection methods primarily rely on histochemical staining, which enables qualitative and semi-quantitative analysis through chromogenic reactions between ROS and specific chemical reagents. For example, 3,3’-diaminobenzidine (DAB) reacts with H_2_O_2_ under peroxidase catalysis to form a brown precipitate, enabling *in situ* localization of H_2_O_2_, while nitroblue tetrazolium (NBT) is reduced by O_2_•^-^ to generate an insoluble blue formazan precipitate (Jambunathan, 2010). These methods are straightforward to implement and cost-effective, providing visual information on ROS accumulation at tissue and subcellular levels. Representative applications include enhanced DAB staining in rice mesophyll cells under salt stress (Jambunathan, 2010). However, these techniques are restricted to specific ROS types and cannot effectively distinguish other reactive species, such as •OH or ¹O_2_. They also depend on subjective interpretation, provide limited quantitative accuracy, and are unsuitable for dynamic monitoring. Although methods such as spectrophotometry, chemiluminescence, and electrochemistry—often based on tissue homogenization—enable quantitative ROS analysis, they compromise cellular integrity and thus fail to reflect the true dynamics of ROS in intact, living plants (Oparka et al., 2016).

### 4.2 Fluorescent probes and gene-encoded sensors

Advances in fluorescence microscopy have established fluorescent probes and genetically encoded sensors as mainstream tools for *in vivo* ROS detection in plants, owing to their high sensitivity, spatiotemporal resolution, and minimal invasiveness.

#### 4.2.1 Small-molecule fluorescent probes

Among small-molecule fluorescent probes, 2′,7′-dichlorodihydrofluorescein diacetate (H_2_DCFDA) is the most extensively used. The parent compound is non-fluorescent but undergoes hydrolysis by intracellular esterases to yield H_2_DCF, which is subsequently oxidized by ROS to form the highly fluorescent product DCF. When coupled with flow cytometry or confocal laser scanning microscopy, this system enables quantitative analysis and visualization of ROS spatiotemporal distribution in model plants such as rice and *Arabidopsis*, with fluorescence intensity showing a strong linear correlation with ROS levels (Ortega-Villasante et al., 2016, 2018).

To enhance specificity and broaden applicability, a variety of functionalized probes have been developed. For instance, the BC-βgal probe, which operates via an intramolecular charge transfer (ICT) mechanism, becomes fluorescent upon β-galactosidase-mediated hydrolysis, enabling specific monitoring of ROS-related enzymatic activity in cabbage roots under heavy metal stress (Cd²^+^, Cu²^+^, Pb²^+^) (Zhao et al., 2023b). Near-infrared (NIR) fluorescent probes have garnered significant interest due to their superior tissue penetration capacity. The DRP-B probe, incorporating a borate ester as the H_2_O_2_ recognition unit, exhibits a turn-on fluorescence signal at 650 nm and has been successfully applied to track H_2_O_2_ fluctuations in cabbage roots subjected to metal toxicity, waterlogging, and drought stresses (Zhao et al., 2023b). Another NIR probe, Cy-Bo, derived from a semi-cyanine fluorophore, emits at 720 nm and has been utilized to visualize H_2_O_2_ accumulation deep within *Arabidopsis* tissues under drought, heat, and salt stress, achieving a detection limit as low as 0.07 μM (Jin et al., 2025). Furthermore, Yin et al. (2022) developed a green fluorescent probe based on a tetraphenylethylene scaffold that permits direct visual monitoring of H_2_O_2_ dynamics under 365 nm UV light, providing a straightforward tool for early phenotypic detection under abiotic stresses including mechanical wounding, high salinity, high light, and drought.

#### 4.2.2 Genetically encoded sensors

Genetically encoded sensors facilitate specific detection and stable monitoring of ROS in living plants by fusing ROS-sensitive proteins—such as transcription factors or peroxidases—with fluorescent proteins (e.g., GFP). These sensors offer the distinct advantage of precise subcellular localization (Nietzel et al., 2019). For example, the HyPer series, derived from a modified *E. coli* OxyR transcription factor, enables real-time monitoring of intracellular H_2_O_2_ levels, although its fluorescence is sensitive to pH fluctuations (Lara-Rojas et al., 2023). To overcome this limitation, the roGFP2-Orp1 sensor was developed by fusing yeast peroxidase Orp1 with redox-sensitive GFP2, enabling pH-insensitive H_2_O_2_ detection. This sensor has been successfully employed to visualize oxidative bursts triggered by excitation stress (Denjalli et al., 2024).

#### 4.2.3 Novel nanosensors

Nanosensors leverage the unique physicochemical properties of nanomaterials—such as high specific surface area and tunable optical characteristics—combined with ROS-sensitive elements to achieve highly sensitive, multi-channel, and even intelligent ROS detection. For instance, the AIENPs@Mo/Cu-POM nanosensor utilizes an aggregation-induced emission (AIE) effect alongside the quenching properties of polyoxometalates (POMs), enabling rapid H_2_O_2_ response (detection limit: 0.43 μM) in the second near-infrared (NIR-II) window at 1035 nm. Notably, when integrated with a machine learning model, this system can effectively distinguish four types of stress—mechanical injury, high temperature, pathogen infection, and high light—with an accuracy exceeding 96.67% (Hu et al., 2025). Other innovative platforms, such as G_3_⊂CP5 and (GT)_15_-DNA-SWNT, further expand capabilities for multi-channel detection and subcellular localization. These platforms enable simultaneous monitoring of signaling molecules like H_2_O_2_ and ATP, and can even quantify the propagation rate of ROS waves under stresses such as mechanical injury, pathogen infection, or high-light stress (Bao et al., 2024; Ang et al., 2024).

### 4.3 Technical trade-offs and integration strategies

While each ROS detection technology offers unique advantages, significant trade-offs exist in specificity, resolution, invasiveness, and applicability. Recognizing these limitations is crucial for selecting appropriate methods and designing robust experimental paradigms. The most promising path forward lies in the strategic integration of complementary technologies.

#### 4.3.1 Key limitations of current technologies

Traditional histochemical methods (e.g., DAB, NBT staining) offer cost-effective *in situ* localization of specific ROS (H_2_O_2_, O_2_•^-^) but are static, qualitative, and unable to distinguish rare species such as •OH or ¹O_2_, with results prone to subjective interpretation (Jambunathan, 2010; Denjalli et al., 2024). Small-molecule fluorescent probes (e.g., H_2_DCFDA, NIR probes like DRP-B and Cy-Bo) exhibit high sensitivity and tissue penetration, but broad-spectrum probes lack ROS species specificity, and functionalized probes may suffer from photobleaching or interference from intracellular redox substances (Ortega-Villasante et al., 2018; Zhao et al., 2023b; Jin et al., 2025). Genetically encoded sensors (e.g., HyPer, roGFP2-Orp1) enable precise subcellular real-time monitoring, yet their application is restricted to transformable species, and some (e.g., HyPer) are susceptible to pH interference (Denjalli et al., 2024). Nanosensors (e.g., AIENPs@Mo/Cu-POM) achieve ultra-high sensitivity and multiplex detection, but face challenges in efficient delivery across plant cell walls (pore size 2–20 nm) and potential long-term biocompatibility issues, with metal-based components possibly inducing unintended ROS production via Fenton reactions (Zhao et al., 2024; Hu et al., 2025). Furthermore, a common trade-off exists between high spatial/temporal resolution and the ability to monitor ROS dynamics over large areas or deep within tissues.

#### 4.3.2 Integrated multi-technology approaches

To overcome the limitations of any single method, integrated approaches that combine technologies are increasingly employed. A powerful strategy couples macroscopic dynamic imaging with microscopic validation, deep-penetrating NIR probes (e.g., Cy-Bo) enable non-invasive monitoring of systemic ROS waves across whole plants (e.g., root-to-shoot propagation under drought), while genetically encoded sensors (e.g., roGFP2-Orp1) confirm the subcellular origin of ROS (e.g., chloroplast PSII) in key regions (Jin et al., 2025; Denjalli et al., 2024). Similarly, qualitative, high-throughput screening (e.g., via DAB staining) can be effectively followed by precise quantitative analysis (e.g., using ratiometric fluorescent probes or nanosensors) on selected samples (Jambunathan, 2010; Hu et al., 2025). Furthermore, integrating imaging data with molecular analyses (e.g., antioxidant enzyme activity assays, stress-related gene expression profiling) constructs a comprehensive evidence chain, linking ROS dynamics to plant physiological responses.

The strategic integration of complementary detection platforms is therefore paramount to transcend the limitations of any single technology. This synergistic approach, exemplified by combining deep-tissue NIR imaging with subcellular-resolution genetically encoded sensors, enables a cross-scale understanding of ROS dynamics, from systemic signaling waves to organelle-specific events. Future technological development must prioritize creating probes with higher specificity (particularly for •OH and ¹O_2_) and photostability, engineering plant-optimized nanosensors with improved delivery and biocompatibility, and building multimodal platforms for simultaneous monitoring of ROS and related signaling molecules (e.g., Ca²^+^). Ultimately, the complementary use of evolving detection tools will continue to refine our spatiotemporal understanding of ROS homeostasis, driving both fundamental discovery and applications in stress-resilient crop breeding.

## 5 Conclusions and outlook

ROS are central regulators of plant growth, development, and adaptation to abiotic stress, exhibiting a dual role as essential signaling molecules and potential agents of oxidative damage. This review has synthesized the stress-specific mechanisms of ROS production, their dual roles in signaling and damage, and the sophisticated multi-layered networks that maintain redox homeostasis, integrating enzymatic/non-enzymatic antioxidants with transcriptional, post-translational, and hormonal regulation.

Accurate patiotemporal monitoring of ROS dynamics is fundamental to understanding these processes. While detection technologies have evolved from static staining to dynamic, quantitative, and *in vivo* imaging, each method presents inherent trade-offs in specificity, resolution, and applicability. As critically discussed in Section 4.3, the path forward lies in the integrative use of complementary platforms (e.g., NIR probes for whole-organ imaging coupled with genetically encoded sensors for subcellular validation) to overcome individual limitations and achieve a cross-scale resolution from macroscopic system signals to microscopic organelle events through the synergistic integration of complementary technologies.

Building upon an integrative research paradigm, future studies should focus on several key frontiers. For example, combining live ROS imaging with multi−omics to systematically decipher the regulatory networks governing ROS homeostasis under stress; developing more specific and photostable probes, plant−compatible nanosensors with enhanced delivery and biodegradability, and multimodal platforms for simultaneous monitoring of ROS and related signaling molecules, for simultaneous monitoring of ROS and key signaling molecules such as Ca²^+^ and pH; applying gene−editing tools to precisely engineer ROS network components for crops that balance stress resilience and growth; and exploring exogenous priming strategies using bio−compatible nanomaterials or compounds to modulate ROS signaling for improved stress adaptation. utilizing gene−editing tools to precisely tailor key components of the ROS network (e.g., RBOH isoforms, antioxidant enzymes) in order to breed crops that balance stress resilience and growth; and exploring the use of bio−compatible nanomaterials or signaling compounds to modulate ROS pathways, thereby providing flexible exogenous strategies for enhancing crop stress adaptation.

In conclusion, the plant ROS research paradigm has comprehensively evolved from an initial focus on “toxic scavenging” to the current emphasis on “precision signaling regulation”. Through interdisciplinary collaboration and deepened investigation into ROS production, perception, decoding, and termination during stress adaptation, scientists will not only advance fundamental understanding of plant stress biology but also establish innovative theoretical and technological frameworks for addressing global climate challenges and ensuring food security.
